# Pet ownership and mental health in United States adults during COVID-19

**DOI:** 10.3389/fpsyg.2023.1217059

**Published:** 2023-10-30

**Authors:** Dolores Marcial-Modesto, Brian N. Chin, Elizabeth D. Casserly, Shelby M. Parsons, Brooke C. Feeney

**Affiliations:** ^1^Department of Psychology, Trinity College, Hartford, CT, United States; ^2^Department of Psychology, Carnegie Mellon University, Pittsburgh, PA, United States

**Keywords:** human-animal relationships, relationship status, depression, anxiety, attachment theory, emotion regulation

## Abstract

The COVID-19 pandemic was associated with declines in mental health and increased interest in pet ownership. We aimed to extend past theories and research linking pet ownership and mental health by investigating whether pet ownership was associated with mental health during the initial phases of the COVID-19 pandemic in a sample of American adults. We also tested whether the association of pet ownership and mental health was moderated by relationship status. Participants were 2,906 American adults who were recruited for an online survey study between May 2020 and May 2021. Pet ownership was assessed via dichotomous self-report (yes/no) and mental health was assessed using a 13-item questionnaire. The sample was 69.2% female with an average age of 46.0 years. 36.1% of the sample owned a pet and 68.5% of the sample was currently partnered. There was no overall association of pet ownership and mental health during the COVID-19 pandemic (estimated mean difference (EMD) = 0.35, 95CI = −0.10, 0.80, *p* = 0.12). However, we found evidence for an association that was moderated by relationship status. Pet ownership was associated with better mental health among partnered individuals (EMD = 0.76, 95CI = 0.21, 1.30, *p* = 0.006). There was no association of pet ownership and mental health among unpartnered individuals (EMD = −0.41, 95CI = −1.20, 0.37, *p* = 0.30). Our findings suggest that relationship status may represent a critical moderator of the link between pet ownership and mental health. Future studies are needed to identify specific mechanisms of pet ownership that could explain its varied impact on the mental health of partnered and unpartnered individuals.

## Introduction

It is well-documented that the COVID-19 pandemic was associated with a worsening of mental health (Bhattacharjee and Acharya, 2020; Ettman et al., 2020). Specifically, the COVID-19 lockdown took a toll on many people’s mental health due to the lack of close social contact, the loss of employment, and the loss of loved ones. In fact, one study conducted shortly after the onset of the COVID-19 pandemic estimated that twice as many people in the United States reported symptoms of depression and anxiety in the months after the onset of the COVID-19 pandemic compared to before the pandemic (Khubchandani et al., 2021).

The early months of the COVID-19 pandemic were also associated with an increase in global interest in pet ownership and pet adoption, including in the United States (Ho et al., 2021). Pandemic-related increases in pet ownership may be attributable to widely reported benefits of pet ownership for humans’ physical health (Dembicki and Anderson, 1996; Jennings, 1997) and mental health (Kurdek, 2008; McConnell et al., 2011; Hui Gan et al., 2020). Companion animals play important roles in modern society, such as serving as emotional support animals, guide and hearing dogs, and event alert dogs that warn people when their blood pressure is high. With reports that nearly 60% of Americans own some type of pet, human-animal relationships are becoming more important to understand than ever (Applebaum et al., 2023).

Research on the beneficial effects of pet ownership has often conceptualized the human-animal bond as a type of attachment relationship. Attachment theory states that humans possess an innate psychobiological system that motivates them to seek proximity to close others during times of stress (Bowlby, 1982). Previous studies indicate that many people may indeed form attachment-like relationships with their pets, and that the beneficial effects of pet ownership on mental health are strongest when humans are more securely attached to their pet (Zilcha-Mano et al., 2012; Teo and Thomas, 2019; Lass-Hennemann et al., 2022).

Other research on the benefits of pet ownership has considered the role of psychological need fulfillment (Kurdek, 2008). Self-determination theory states that close relationships promote well-being by fulfilling the basic psychological needs of autonomy, competence, and relatedness (Ryan and Deci, 2000). Kanat-Maymon et al. (2016) found that pet owners’ perceived needs supported by their pet predicted higher well-being above and beyond the needs supported by their close human relationships. Thus, it is plausible that the beneficial effects of pet ownership on mental health may be driven by the fulfillment of psychological needs.

Although theories of pet ownership and mental health tend to focus on the benefits of pet ownership, past studies comparing the mental health of pet owners and non-owners have provided conflicting evidence for this hypothesis. Scoresby et al. (2021) recently conducted a systematic review of 41 studies examining pet ownership and mental health. Their review found 17 studies suggesting a positive impact of pet ownership on mental health, 5 studies suggesting a negative impact of pet ownership on mental health, 13 studies suggesting no impact of pet ownership on mental health, and 19 studies suggesting a mixed impact of pet ownership on mental health.

Studies conducted during the COVID-19 pandemic have similarly suggested a mixed impact of pet ownership on mental health. A cross-sectional study of Singaporean adults by Tan et al. (2021) reported that pet ownership was associated with better emotional well-being; this study also reported a positive association of pet attachment and emotional well-being among pet owners. Barklam and Felisberti (2023) reported a mixed impact of pet ownership on mental health in their study conducted during the initial phases of the COVID-19 pandemic in an international sample. Specifically, pet ownership was positively associated with mental health for individuals with low resilience but negatively associated with mental health for individuals with high resilience. These researchers suggested that individuals reporting higher resilience in this context may be more likely to suffer from maladaptive self-enhancement biases that lead to difficulties with social and psychological functioning (Bonanno et al., 2005). A longitudinal study of Canadian adults by Falck et al. (2022) reported that pet ownership was associated with *poorer* mental health among individuals with a diagnosed mental health condition; pet ownership was not associated with mental health among individuals without a mental health condition. This study suggested that pet owners with mental health conditions may have experienced additional burden from the challenges of caring for a pet during a pandemic (e.g., obtaining food and supplies and accessing veterinary care).

The current study aimed to test whether pet ownership was associated with mental health during the COVID-19 pandemic (Aim 1), and whether the association between pet ownership and mental health was moderated by individual differences in relationship status (Aim 2). We hypothesized that pet owners would report better mental health than non-owners during the COVID-19 pandemic (Hypothesis 1). We also hypothesized that the effect of pet ownership on mental health would be moderated by individual differences in relationship status; however, we did not make specific hypotheses regarding the nature of these moderated effects.

## Method

### Participants and procedures

Participants were 2,906 United States adults who were recruited via online advertisements for a larger study of the COVID-19 pandemic’s impact on relationships and well-being. Inclusion criteria were being 18+ years old and fluent in English. Data were collected from May 2020 to May 2021. Study procedures were approved by the Carnegie Mellon University institutional review board (Number: STUDY2020_00000178, Title: Social Impact of COVID-19 Study). After providing informed consent, participants completed questionnaires assessing their demographic information (age, gender, race/ethnicity, educational attainment, region, relationship status, parenthood status), pet ownership, and mental health as part of a larger investigation of social experiences during the COVID-19 pandemic. Participants did not receive compensation for completing the study.

#### Power analysis

The sample size for the parent study was determined based on the desire to maximize power by collecting data from as many participants as possible. We conducted an *a priori* power analysis using G*Power (Faul et al., 2007) to determine the sample size needed to test Aim 1. Specifically, our power analysis tested the number of participants needed to detect a medium-sized effect (*f* = 0.25) and a small-sized effect (*f* = 0.10) using an analysis of covariance with an error probability of *ɑ* = 0.05, desired power of 0.95, two groups, and 11 covariates. The required total sample size was *N* = 210 for a medium effect and *N* = 1,302 for a small effect.

### Measures

#### Pet ownership

Participants were asked to indicate whether they currently lived with anyone during the pandemic with the response options of: people only, pets only, people and pets, I live alone. Participants were categorized as pet owners if they endorsed living with *pets only* or with *people and pets*.

#### Mental health

Participants rated 13 items assessing the extent to which they had been experiencing symptoms of depression and anxiety since the start of the COVID-19 pandemic on a scale from 1 (*not at all*) to 3 (*very much*): feeling depressed, feeling nervous or anxious, feeling hopeless, feeling sad or blue, feeling angry or hostile, could not focus, sleep disturbances, difficulty thinking clearly, feeling that your heart is racing, feeling stressed, feeling discouraged, ruminating about worries, feeling rejected. These items were selected based on their face validity, their common factor loading, and their similarity to items from previously validated measures of mental health symptoms, including several items that were adapted from the Center for Epidemiological Studies Depression Scale (Radloff, 1977) and the Generalized Anxiety Disorder 7 (Spitzer et al., 2006). A single composite variable was computed by summing the scores of the 13 items (*ɑ* = 0.92). Finally, we reverse-scored this composite, so higher scores indicated better mental health.

#### Relationship status

Participants indicated their current relationship status: single, in a committed relationship, engaged, married, common law marriage, separated, divorced, widowed, or other. These responses were used to categorize participants as being *partnered* (in a committed relationship, engaged, married, common law marriage) or *unpartnered* (single, divorced, widowed, separated). Participants were excluded from analysis if they responded other or if they preferred not to disclose their relationship status (*n* = 59).

### Data analysis

Analyses were conducted using SPSS software (Version 28.0, IBM, Armonk, NY, USA) using the UNIANOVA command.

We investigated Aim 1 by conducting a one-way analysis of covariance to examine whether pet ownership (yes/no) was associated with mental health during the COVID-19 pandemic when controlling for age, gender, race/ethnicity, educational attainment, and parenthood status. Pairwise comparisons were used to evaluate the significance of between-group differences (pet owner/non-owner) based on the estimated marginal means and standard errors.

We investigated Aim 2 by conducting a two-way analysis of covariance to examine the interaction of pet ownership (yes/no) and relationship status (partnered/unpartnered) on mental health during the COVID-19 pandemic when controlling for age, gender, race/ethnicity, educational attainment, and parenthood status. We investigated statistically significant interactions by using pairwise comparisons to evaluate the significance of between-group differences (pet owner/non-owner) based on the estimated marginal means and standard errors in pet owners and non-owners.

## Results

### Preliminary analyses

The demographic characteristics of the final sample are summarized in Table 1. The average participant was 46.0 years old (*SD* = 17.2 years). The sample primarily consisted of individuals who were female, White, and had attained at least a bachelor’s degree. 36.1% of the sample owned a pet and 68.5% of the sample was currently partnered. Mental health scores ranged from 1 to 39 with a mean of 16.67 (*SD* = 6.52) in the overall sample.

**Table 1:** Demographic characteristics of the full sample and stratified by pet ownership.

	Full sample (*N* = 2,906)	Pet owners (*N* = 1,048)	Non-owners (*N* = 1858)	*p* diff
Age, years	46.0 (17.2)	45.4 (18.2)	46.4 (16.6)	0.14
Gender, % (*n*)				0.28
Female	69.2 (2010)	67.1 (703)	70.3 (1307)	
Male	28.5 (828)	30.6 (321)	27.3 (507)	
Non-binary	2.1 (61)	2.1 (22)	2.1 (39)	
Prefer not to disclose	0.2 (7)	0.2 (2)	0.3 (5)	
Race, % (*n*)				<0.001
White	86.4 (2512)	79.3 (831)	90.5 (1681)	
Black or African American	3.2 (94)	5.6 (59)	1.9 (35)	
Asian or Asian American	5.3 (153)	7.3 (77)	4.1 (76)	
Hispanic or Latin (x)	4.8 (139)	5.1 (53)	4.6 (86)	
American Indian or Alaska Native	3.2 (94)	4.9 (51)	2.3 (43)	
Native Hawaiian or Pacific Islander	0.5 (14)	0.3 (3)	0.6 (11)	
Other race	1.8 (52)	1.3 (14)	2.0 (38)	
Prefer not to disclose	1.1 (33)	1.0 (10)	1.2 (23)	
Relationship status, % (*n*)				0.003
Single	19.2 (558)	22.7 (238)	17.2 (320)	
Partnered	68.5 (1992)	66.4 (696)	69.8 (1296)	
Divorced, widowed, or separated	10.2 (297)	9.1 (95)	10.9 (202)	
Other/prefer not to disclose	2.0 (59)	1.8 (19)	2.2 (40)	
Region, % (*n*)				0.024
South	27.8 (807)	25.8 (270)	28.9 (537)	
Northeast	25.1 (728)	28.1 (295)	23.3 (433)	
Midwest	19.6 (570)	18.7 (196)	20.1 (374)	
West	27.2 (791)	26.9 (282)	27.4 (509)	
Prefer not to disclose	0.3 (10)	0.5 (5)	0.3 (5)	
Educational attainment, % (*n*)				0.48
Did not complete high school	0.6 (16)	0.6 (6)	0.5(10)	
Completed high school	4.4 (128)	5.4 (57)	3.8 (71)	
Some college	14.7 (426)	14.6 (153)	14.7 (273)	
Associate’s degree	7.5 (219)	7.3 (76)	7.7 (143)	
Bachelor’s degree	29.6 (861)	29.2 (306)	29.9 (555)	
Some graduate school	7.4 (216)	7.1 (74)	7.6 (142)	
Master’s degree	24.6 (715)	23.7 (248)	25.1 (467)	
Professional degree	10.6 (308)	11.5 (121)	10.1 (187)	
Prefer not to disclose	0.6 (17)	0.7 (7)	0.5 (10)	
Parenthood status, % (*n*)				0.12
No children at home	71.3 (2072)	70.3 (1306)	73.1 (766)	
Children at home full- or part-time	28.5 (827)	29.4 (547)	26.7 (280)	
Prefer not to disclose	0.2 (7)	0.3 (5)	0.2 (2)	

Age differences were evaluated using an independent-samples *t*-test with equal variances not assumed. Gender, race, relationship status, region, educational attainment, and parenthood status differences were evaluated using chi-squared tests.

### Aim 1: association of pet ownership and mental health

**Figure 1:**
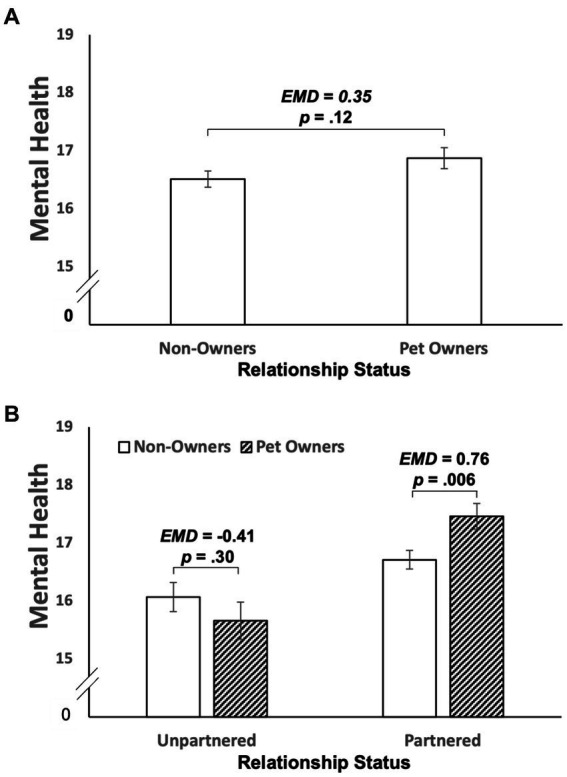
Association of pet ownership and mental health in the overall sample **(A)** and separately in partnered and unpartnered participants **(B)**. This figure shows the estimated marginal mean mental health scores by pet ownership when controlling for age, gender, race/ethnicity, educational attainment, and parenthood status. Between-group differences were tested using the estimated mean differences (EMD).

We tested whether pet ownership (pet owner/non-owner) was associated with mental health during the COVID-19 pandemic when controlling for age, gender, race/ethnicity, educational attainment, and parenthood status. As shown in Figure 1A, the estimated marginal mean mental health score was 16.87 for pet owners (*SE* = 0.18) and 16.51 for non-owners (*SE* = 0.14). Contrary to Hypothesis 1, pet owners did not report higher mental health scores than non-owners (estimated mean difference = 0.35, 95CI = −0.10, 0.80, *p* = 0.12).

### Aim 2: moderated association of pet ownership and mental health

We tested whether the association of pet ownership and mental health during the COVID-19 pandemic was moderated by individual differences in relationship status (partnered/unpartnered) when controlling for age, gender, race/ethnicity, educational attainment, and parenthood status. Consistent with our hypothesis, we observed an interaction of pet ownership x relationship status on mental health scores (*p* = 0.017).

To probe this interaction, we conducted pairwise comparisons to test between-group differences (pet owner/non-owner) in mental health scores among partnered and unpartnered individuals. As shown in Figure 1B, the estimated marginal mean mental health score was 15.66 (*SE* = 0.32) for unpartnered pet owners, 16.07 (*SE* = 0.25) for unpartnered non-owners, 17.46 (*SE* = 0.22) for partnered pet owners, and 16.71 (*SE* = 0.16) for partnered non-owners. Partnered pet owners reported higher mental health scores than partnered non-owners (estimated mean difference = 0.76, 95CI = 0.21, 1.30, *p* = 0.006). However, unpartnered pet owners did not report higher mental health scores than unpartnered non-owners (estimated mean difference = −0.41, 95CI = −1.20, 0.37, *p* = 0.30).

## Discussion

This observational study evaluated the association of pet ownership and mental health during the COVID-19 pandemic and examined relationship status as a potential moderator of this association. Pet ownership was associated with better mental health during the COVID-19 pandemic but only among partnered individuals; unpartnered individuals did not experience mental health benefits from pet ownership. These results are partially consistent with existing theories of pet ownership that primarily focus on the mental health and well-being benefits of owning a pet (Bowlby, 1982; Kurdek, 2008; Zilcha-Mano et al., 2012). They add to growing evidence for mixed effects of pet ownership on mental health during the initial phases of the COVID-19 pandemic (Amiot et al., 2022; Falck et al., 2022; Barklam and Felisberti, 2023) by identifying relationship status as a potentially critical sociodemographic moderator of this effect.

There are several possible explanations for why we did not observe an *overall* association of pet ownership and mental health during the COVID-19 pandemic. First, it is possible that the beneficial effects of pet ownership on mental health were mitigated by the additional challenges of owning a pet during a pandemic, such as the need to obtain food, supplies, and veterinary care, and the changes to owners’ and pets’ routines caused by working or attending school from home during this time (Applebaum et al., 2020). These challenges may have been especially burdensome for individuals with preexisting mental health conditions (Falck et al., 2022). However, we could not test this proposed explanation because our study did not assess whether participants had a diagnosed mental health condition before the start of the pandemic. Second, it is possible that the beneficial effects of pet ownership on mental health are driven by owning specific types of pets. For example, Oliva and Johnston (2021) found that dog ownership – but not cat ownership – was protective against loneliness during the COVID-19 lockdown in Australian adults who lived alone. Third, it is possible that the beneficial effects of pet ownership may depend on other unexamined factors, such as the strength of one’s attachment to their pet(s) and/or other people (Teo and Thomas, 2019; Lass-Hennemann et al., 2022). These effects may also depend on how recently a pet was acquired – a factor that is especially salient given the high rates of pet adoption during the COVID-19 pandemic (Ho et al., 2021).

Our findings suggest that the association of pet ownership and mental health is more complex than initially believed and may depend on sociodemographic factors. This observation is consistent with a recent systematic review of studies of pet ownership and mental health which found the strongest evidence for mixed effects of pet ownership on well-being (Scoresby et al., 2021). It is also consistent with other studies conducted during the COVID-19 pandemic which have found a mixed impact of pet ownership on mental health (Falck et al., 2022; Barklam and Felisberti, 2023). Why did pet ownership benefit mental health for partnered individuals only? We believe that a compelling explanation for this finding is that partnership impacts the cost–benefit ratio of pet ownership. For example, it is possible that sharing pet ownership with a romantic partner reduces the costs of pet ownership (e.g., caregiving responsibilities; daily feeding and grooming; veterinary care) while maintaining or perhaps enhancing the benefits of pet ownership (e.g., companionship and attachment; psychological sense of purpose and identity). It is also possible that it was less burdensome for pet owners to navigate the challenges of owning a pet during a pandemic if they had the support of a partner (Applebaum et al., 2020). Another possible explanation is that pet ownership may be indicative of better relationship quality in partnered individuals. Indeed, earlier studies have found that couples with pets tend to report greater relationship satisfaction than couples without pets (Cloutier and Peetz, 2016). Future studies are needed to test these proposed explanations.

Strengths of this investigation include its use of data that were collected during a worldwide pandemic from a large sample of nearly 3,000 United States adults. There are also certain limitations of this study, which we hope will be addressed by future research. First, a significant limitation of this work is that our mental health scale was adapted from existing measures but has not been previously validated. Future investigations are needed to test whether these associations are replicable when mental health is assessed using previously validated and reliable measures of depression and anxiety symptoms. Second, we tested our hypotheses using a dichotomous measure of pet ownership and did not focus on the role of specific pet ownership characteristics, such as the number or type of pet(s) owned, the strength of attachment to one’s pets, whether the participant serves as their pet’s primary caregiver, or whether the pet was already owned before the pandemic or adopted after the pandemic’s onset. Third, our sample primarily consisted of white and female participants which could limit the external validity of our findings. Future studies should address this issue by continuing to explore the sociodemographic and personality factors that moderate the effects of pet ownership on health and well-being. Finally, these data were cross-sectional which limits our ability to make a causal inference about the association of pet ownership and mental health. We acknowledge the possibility of reverse-causation given that pet ownership may be influenced by one’s mental health and relationship status. Future studies should continue to investigate the issue of causality by collecting longitudinal data and evaluating plausible mechanisms through which the human-pet relationship benefits mental health. Candidate mechanisms include promoting positive health behaviors (physical activity, sleep, leisure activity, daily routines), discouraging risky health behaviors (smoking, alcohol consumption, and substance use), and reducing psychobiological stress reactivity.

This study is the first to identify relationship status as a critical sociodemographic factor that moderates the association between pet ownership and mental health. While pet ownership was associated with better mental health for partnered individuals, the mental health benefits of pet ownership were not observed among unpartnered individuals. These findings add to growing evidence for the mixed effects of pet ownership on mental health, especially during the initial phases of COVID-19 pandemic. Future research is needed to elucidate the potentially synergistic role of human and animal relationships in regulating mental health.

## Data availability statement

The raw data supporting the conclusions of this article will be made available by the authors, without undue reservation.

## Ethics statement

The studies involving human participants were reviewed and approved by Carnegie Mellon University’s Institutional Review Board. The patients/participants provided their written informed consent to participate in this study.

## Author contributions

BF designed and executed the parent study. DM-M, BC, and EC conceptualized the research question and hypotheses presented in this manuscript. DM-M and BC conducted the statistical analyses. DM-M drafted the manuscript with support from BC. All authors contributed to interpretation of the results, provided critical feedback on drafts of the manuscript and approved the final version.
